# Autonomous navigation and control of magnetic microcarriers using potential field algorithm and adaptive non-linear PID

**DOI:** 10.3389/frobt.2024.1439427

**Published:** 2024-08-13

**Authors:** Mohamed Sallam, Mohamed A. Shamseldin, Fanny Ficuciello

**Affiliations:** ^1^ ICAROS Lab, Department of Electrical Engineering and Information Technology, University of Naples Federico II, Naples, Italy; ^2^ Department of Mechanical Engineering, Helwan University, Cairo, Egypt; ^3^ Department of Mechatronics Engineering, Egyptian Chinese University, Cairo, Egypt

**Keywords:** magnetic control, drug delivery, path planning, paramagnetic microparticle, ANPID

## Abstract

Microparticles are increasingly employed as drug carriers inside the human body. To avoid collision with environment, they reach their destination following a predefined trajectory. However, due to the various disturbances, tracking control of microparticles is still a challenge. In this work, we propose to use an Adaptive Nonlinear PID (A-NPID) controller for trajectory tracking of microparticles. A-NPID allows the gains to be continuously adjusted to satisfy the performance requirements at different operating conditions. An *in-vitro* study is conducted to verify the proposed controller where a microparticle of 100 μ m diameter is put to navigate through an open fluidic reservoir with virtual obstacles. Firstly, a collision-free trajectory is generated using a path-planning algorithm. Secondly, the microparticle dynamic model, when moving under the influence of external forces, is derived, and employed to design the A-NPID control law. The proposed controller successfully allowed the particle to navigate autonomously following the reference collision-free trajectory in presence of varying environmental conditions. Moreover, the particle could reach its targeted position with a minimal steady-state error of 4 μ m. A degradation in the performance was observed when only a PID controller was used in the absence of adaptive terms. The results have been verified by simulation and experimentally.

## 1 Introduction

Microrobots have considerable potential to revolutionize the treatment of several diseases that are currently considered difficult to cure (Nelson et al., 2010). Due to their small size, they can navigate through hard-to-access regions inside the human body, and precisely deliver drug doses to target lesions in a minimal-invasive and effective manner Abbott et al. (2007); Sitti (2009); (Nelson et al., 2010); Khalil et al. (2012a); Scheggi and Misra (2016). This precision delivery means that a higher concentration of the drug will arrive at the most beneficial site, and that the risk of potential side effects is minimized because the drug is much less likely to diffuse to the surrounding tissue. However, to reach their destination, microparticles have to navigate through a complex network of blood vessels with multiple forks and narrow paths Belharet et al. (2012); Coene et al. (2015). Steering the particle in such environment can be realized by means of a teleoperation device i.e. joystick which allows the operator to remotely guide the particle towards the targeted goal. The operator can get his feedback about the particle position through an artificial image reconstructed from the slices obtained from MRI. However, this procedure is very difficult and stressful even for a skilled operator, mostly because of the reduced workspace, the high precision required, the lack of haptic perception and the complexity induced by artificial vision feedback. Alternatively, closed-loop control system allows microparticles to autonomously reach their target position following an obstacle-free trajectory without the intervention of the operator. For this purpose, a path planning algorithm has to be firstly used to generate a reference trajectory that avoids the collision with the surrounding environment.Several path-planning algorithms have been presented in the literature for autonomous navigation of mobile robots such as A^*^ algorithm Lin et al. (2017), Dijkstra algorithm Wang et al. (2011), RRT algorithm Gong et al. (2014), probabilistic roadmaps (PRM) Amato and Wu (1996); Kavraki et al. (1996), genetic algorithm Liu et al. (2004), ant colony algorithm Wang et al. (2018), and artificial potential field algorithm Qi et al. (2008). However, navigation inside the human body is more challenging task as several constraints and physiological issues have to be considered. For instance, the path planning algorithm must ensure that the ratio between the diameter of the microparticle and the diameter of blood vessel satisfies a specific range, and the microparticle can counteract the reciprocal blood flow which is typically higher in larger diameter blood vessels Sabra et al. (2005).

A comparative study was conducted in Sabra et al. (2005) between a group of path planning algorithms to find the best trajectory to be followed by a microparticle to reach a goal in the cardiovascular system. The study took into consideration the exclusive features of the blood circulatory system beside the other constraints that are associated with the navigation inside the human body. The criteria for the best algorithm were computation time, memory usage, local minima handling, and the capability of determining multiple paths. Among nine algorithms, the Artificial Potential Filed (APF) was found one of the most appropriate. Recently, another experimental comparison between six path planning algorithms when applied to the motion control of paramagnetic microparticles was presented in Scheggi and Misra (2016). The comparison was conducted based on three metrics i.e., computation time, trajectory length, and elapsed time. The experimental results revealed equivalence between almost all the considered planners in terms of trajectory length and completion time while the artificial potential field and A^*^ with quadtree achieved the best performances regarding the computation time. The APF algorithm was also presented in other several studies as in Khalil et al. (2012a) where an untethered microrobot was wirelessly controlled to reach its destination with obstacle avoidance. Motivated by the satisfactory performance of the APF algorithm in the previous studies, it is selected in this work to generate a collision-free path for the microparticle while flowing in a an open fluidic resvoir with virtual narrow vessel-like channel and static obstacles.To ensure accurate trajectory tracking of the microparticle, a closed-loop control system is indispensable. Several control logarithms have been presented in the literature for the position tracking of magnetically actuated microparticles. For instance, in Khamesee et al. (2002) the PID controller was applied to the microrobot position, but its performance was unsatisfactory as practically, the microrobot is a complicated system with high nonlinearity and uncertainty. In addition, at microscale, the external disturbances and dynamic uncertainties may have a greater influence on the motion of microparticles Jiang et al. (2022). This makes classic linear controllers like PID inadequate for such a control task. Thus, more advanced model-based nonlinear control strategies have been reported to improve the position tracking performance Piepmeier et al. (2014); Mellal et al. (2016). However, their performance were still insufficient to achieve position tracking due to the lack of accurate mathematical models describing the dynamic effects Zhang et al. (2013). Other adaptive, robust and optimal control algorithms could demonstrate ability to respect the performance measures under high model uncertainties and environmental disturbances Marino et al. (2014); Ma et al. (2017); Meng et al. (2020).

In this work, an adaptive-nonlinear PID control algorithm is proposed for trajectory tracking of microparticles. The proposed controller allows the gains to be adjusted online in order to satisfy the performance requirements at different operating conditions Das and Sengupta (2018), Li and Yuan (2020). The A-NPID algorithm has been used in the literature with various applications but to the best of our knowledge it has never been tested with micro-sized agents which exhibit high sensitivity to environmental variables Liu et al. (2020); Rithirun et al. (2021). Moreover, the paramagnetic microparticles employed in this research are magnetically driven in a very small workspace adding an additional challenge due to the water concave meniscus formed near the wall of the reservoir. The proposed controller is investigated through conducting a complete study that aims to ensure successful navigation and control of a microparticle flowing in an open fluidic resvoir with virtual narrow vessel-like channel and static obstacles. As such, in this study, we achieve the following.

• Employing a path planning algorithm (i.e., Artificial Potential Field) to generate a collision-free trajectory for the navigation of the microparticle inside a virtual fluidic channel with static obstacles.

• Deriving a mathematical model that describes the motion of the microparticle when flowing in water under different types of forces i.e., magnetic force and drag force.

• Development of a closed-loop control system based on adaptive-nonlinear PID algorithm that allows for effective trajectory tracking in presence of the environmental disturbances and uncertainties.

• Evaluating the proposed motion control strategy experimentally and by simulation.

The remainder of this article is organized as follows: Section 2 presents the description of the electromagnetic system and its dynamic model derivation. Section 3 presents the development of the Artificial Potential Field path planning algorithm. Section 4 presents the implementation of the adaptive-nonlinear PID controller in addition to the simulation results. Section 5 presents the experimental verification. Finally, section 6 concludes this article and provides directions for future work.

## 2 Materials and Methods

### 2.1 System description

**FIGURE 1:**
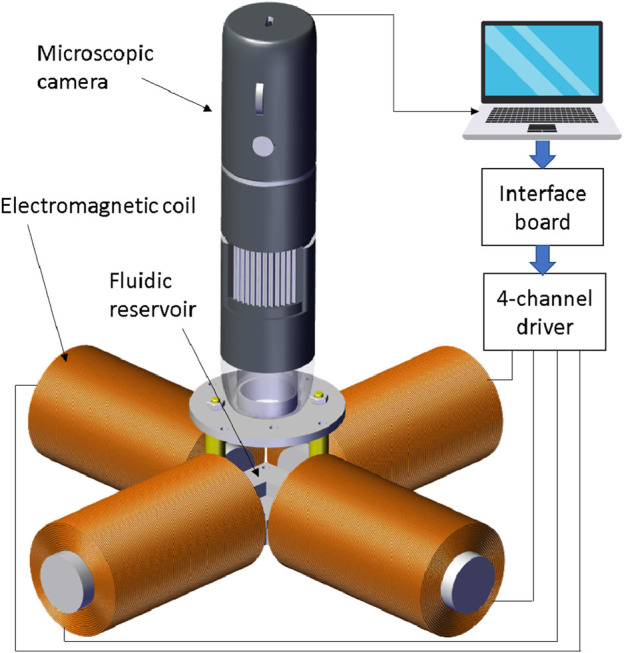
Electromagnetic system for autonomous navigation and control of microparticles. The setup consists of four lateral coils placed in a symmetrical perpendicular configuration. A microscopic camera is positioned properly above the workspace to detect the particle position.

**FIGURE 2:**
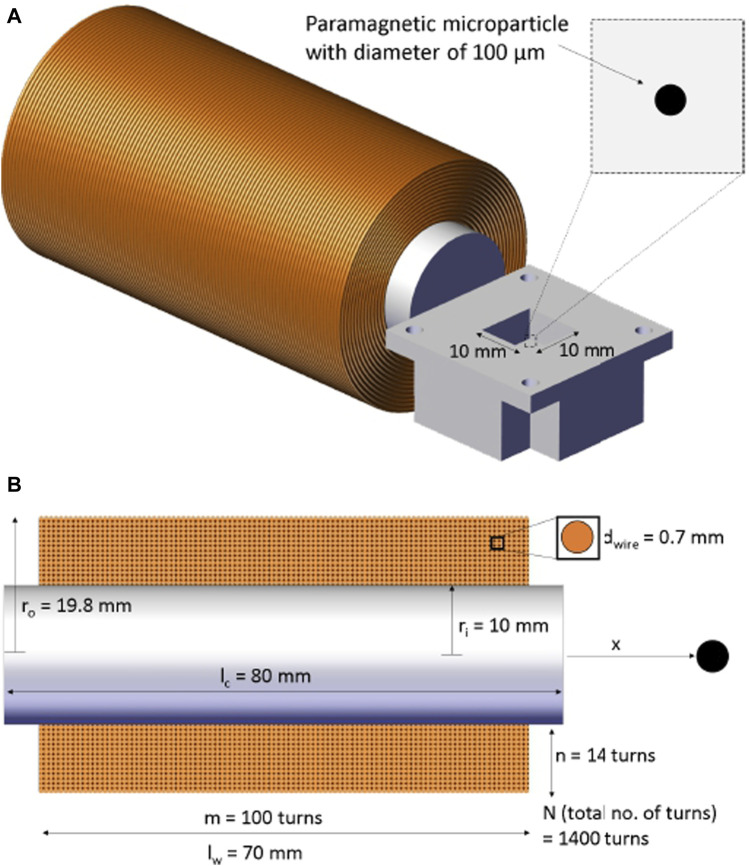
Schematic of the employed electromagnetic coil showing its dimensions and number of turns. **(A)** The microparticle is allowed to move within a workspace of 10 × 10 mm yet the region of interest is only about 3 × 3 mm. **(B)** A cross-section view of the coil shows number of turns in lateral and axial directions.

The system consists mainly of four equally sized coils with metal cores used to generate the magnetic field needed to move the particle (see Figure 1). Each coil has 1,400 turns of 0.7 mm round copper wire coated with enamel. The inner radius of the coil $\left(r_{i}\right)$ is 10 mm, the outer radius $\left(r_{o}\right)$ is 19.8 mm, the length of the core $\left(l_{c}\right)$ is 80 mm, and the axial length of the windings $\left(l_{w}\right)$ is 70 mm. A schematic view of the coil is shown in Figure 2A. The coil cores are made of ferromagnetic material i.e., low carbon steel with relative permeability $\mu_{r}$ of around 100 Figure 2B. Many researchers in the literature prefer employing electromagnets with air-core rather than with ferromagnetic core to avoid the effect of magnetic hysteresis. Air-core allows having a linear relation between the input current to the coils and generated magnetic field. However, due the low permeability of the air, the generated magnetic field is rather weak which may restrict having a precise motion control inside the human body where the particle has to flow in blood-vessels with high flow-rate. On the other side, although ferromagnetic cores have high permeability allowing strong magnetic field, they cause the relation between the input current and generated magnetic field to be non-linear due to the magnetic hysteresis. It is expected that due to the small workspace, the effect of the hysteresis will be at its minimum. Moreover, the proposed adaptive non-linear controller should be able to cope with such non-linear dynamics due the hysteresis.

The average series resistance of each coil is measured to be around 6 Ω . The dimensions of the fluidic reservoir are selected to be 10 mm × 10 mm. However, the region of interest is about 3 mm × 3 mm to be viewable underneath the microscope, and also to have less effect of the surface tension near the edges of the reservoir. The microparticle is planned to be floating in the water-to-air boundary layer (see Figure 2A). We are using paramagnetic spherical microparticles, consisting of iron-oxide in a poly(lactic acid) matrix (PLAParticles-M-redF-plain from Micromod Partikeltechnologie GmbH, Rostock-Warnemuende, Germany) with an average diameter of 100 μ m. The employed paramagnetic particle was selected over other types of ferromagnetic microparticles to minimize the effect of magnetization and hystresis. As known, paramagnetic materials become magnetized in a magnetic field but their magnetism disappears when the field is removed. On the other side, ferromagnetic materials can retain their magnetic properties when the magnetic field is removed. The particle motion is detected by a 1,000x digital microscopic camera that is positioned properly above the target. The acquired images are processed and the particle position is detected using the vision-assistant toolbox of Labview. A computer-based control is adopted to have the sufficient power of processing the acquired images simultaneously with running the A-NPID control algorithm in real-time. The input provided to the controller is the difference between the current and desired position of the particle. The output of the controllers is used to set the current through the coils.

In order to reduce the coupling effect between the coils, the current direction in all coils should be the same. Having different directions of the current showed that the coupling effect significantly reduces the generated magnetic field.

### 2.2 Motion equation

Microparticles move in the fluid under the influence of two main forces; the external magnetic force, and drag force. Firstly, the formula of each of these two forces are found. Then, the equation that governs the motion of the particle is derived.

The magnetic force $\left(F_{m}\right)$ exerted on a paramagnetic microparticle can be calculated using the following equation Mathieu and Martel (2010):

$$F_{m}=\nabla \left(m\cdot B\right)$$ (1)

where m is the magnetic moment of the particle and B is the applied magnetic field. For a paramagnetic microparticle, m can be expressed as,

$$m=\frac{\chi_{m}}{\mu }V_{\text{p}}B$$ (2)

where $V_{\text{p}}$ is the volume of the particle, $\chi_{m}$ is the magnetic susceptibility constant McNeil et al. (1995) [ $\chi_{m}=0.17$ for our magnetic microparticle Khalil et al. (2012b)], and μ is the permeability coefficient given by $\mu_{0}\left(1+\chi_{m}\right)$ . Furthermore, $\mu_{0}$ is the permeability of vacuum $\left(\mu_{0}=4\pi \times 10^{-7}T.m/A\right)$ . Assuming the particle has a perfect spherical shape with a radius $r_{p}$ , then $V_{\text{p}}=\frac{4}{3}\pi r_{p}^{3}$ . By substitute in Equation 2 yields

$$m=\frac{4}{3}\pi r_{p}^{3}\frac{\chi_{m}}{\mu_{0}\left(1+\chi_{m}\right)}B$$ (3)

Combining Equation 1 and Equation 3 results in:

$$F_{m}=\frac{4}{3}\pi r_{p}^{3}\frac{\chi_{m}}{\mu_{0}\left(1+\chi_{m}\right)}\nabla \left(B^{2}\right)$$ (4)

The magnetic field B is linearly proportional to the applied current I . If we consider the contribution of a single electromagnet on the particle assuming the magnetic field has only one component in the axial direction of the coil, then the magnetic field can be expressed as following:

$$B=\left(B_{x}\right)\hat{x}=\tilde{B}I$$ (5)

where $B_{x}$ is the x-component of the magnetic field i.e. the axial direction of the coil, $\tilde{B}$ is a one-dimensional vector with a magnitude that depends on the distance at which the magnetic field is measured and I is a scalar value of the applied current. Therefore, $\nabla \left(B^{2}\right)$ can be computed using the magnetic field gradient as follows:

$$\nabla \left(B^{2}\right)=\frac{\partial {\tilde{B}}^{2}}{\partial x}I^{2}$$ (6)

Substituting Equation 6 in Equation 4 yields

$$F_{m}=\frac{4}{3}\pi r_{p}^{3}\frac{\chi_{m}}{\mu_{0}\left(1+\chi_{m}\right)}\frac{\partial {\tilde{B}}^{2}}{\partial x}I^{2}$$ (7)

**FIGURE 3:**
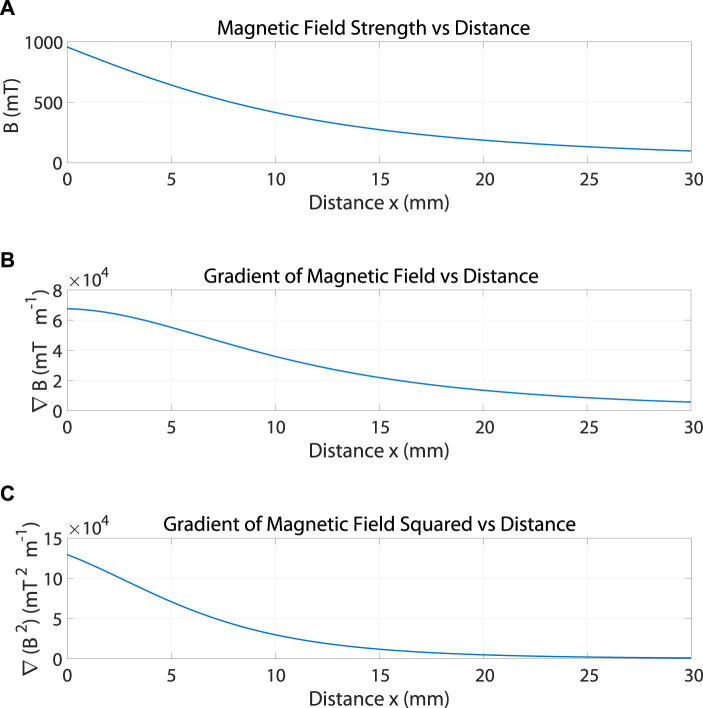
**(A)** The magnetic field B generated by the coil in the axial direction due to the flowing of DC current of 0.8 A. **(B)** The gradient of the magnetic field ∇(B) . **(C)** The gradient of the magnetic field squared $\nabla \left(B^{2}\right)$ .

Assuming the windings of our coil are perfectly stacked, the field-current relation can be theoretically found by the following formula:

$$B=\left(\frac{\mu_{0}\mu_{r}}{2}\overset{m}{\underset{i=1}{\sum }}\overset{n}{\underset{j=1}{\sum }}\frac{{\left(r_{s}+j*d_{\text{w}}\right)}^{2}}{{\left({\left(r_{s}+j*d_{\text{w}}\right)}^{2}+{\left(x+i*d_{\text{w}}\right)}^{2}\right)}^{\frac{3}{2}}}\right)I$$ (8)

where x is the distance from the side of the coil to a point on the axis, $r_{s}$ is the radius of the smallest winding on the axis and $d_{w}$ is the diameter of the wire Figure 2B. The iterator j represents the number of windings in the radial direction and iterator i represents the number of windings in the axial direction. Figure 3A shows the magnetic field B generated by the coil in the axial direction due to the flowing of DC current of 0.8 A using the formula of Equation 8. Figures 3B, C show the gradient of the magnetic field ∇(B) and the gradient of the magnetic field squared $\nabla \left(B^{2}\right)$ respectively.

**FIGURE 4:**
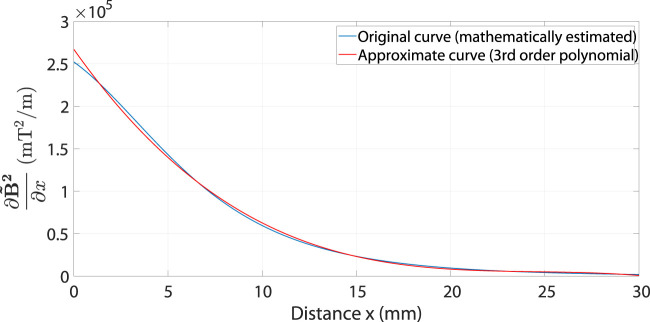
The estimated gradient of the squared magnetic field in *x* direction is represented using a polynomial of third order system obtained from curve fitting operation. $\frac{\partial {\tilde{B}}^{2}}{\partial x}=-17.15x^{3}+1264x^{2}-31360x+267100$

The term multiplied by the current I in Equation 8 is equivalent to $\tilde{B}$ in Equation 5. This term is firstly used to find $\frac{\partial {\tilde{B}}^{2}}{\partial x}$ at different values of x . Then, the obtained results are imported into the curve fitting tool box of MATLAB to be represented by an equivalent polynomial. A satisfactory representation could be achieved by a 3rd order polynomial (see Equation 9). Figure 4 shows the result of the curve fitting operation.

$$\tilde{f\left(x\right)}=\frac{\partial {\tilde{B}}^{2}}{\partial x}=-17.15x^{3}+1264x^{2}-31360x+267100$$ (9)

Substituting Equation 9 in Equation 7 yields

$$F_{m}=\frac{4}{3}\pi r_{p}^{3}\frac{\chi_{m}}{\mu_{0}\left(1+\chi_{m}\right)}\tilde{f\left(x\right)}I^{2}$$ (10)

It can be shown from Equation 10 that the generated force is a function of the microparticle size and geometry, the distance between the particle and the coil, and the applied current. The force current map Equation 10 is used to determine whether the generated magnetic force would overcome the viscous drag force $\left(F_{d}\right)$ generated due to the motion of the microparticle inside the fluid. In order to determine the drag force on the particle, we firstly determine the Reynolds number, Equation 11

$$Re=\frac{2\rho vr_{\text{p}}}{\eta }$$ (11)

where v , η and ρ are the microparticle velocity, fluid dynamic viscosity (1 mPa.s ) and density (998.2 $kg/m^{3}$ ), respectively. Assuming that v will not exceed 1 mm/s, Reynolds number turns out to be less than 0.1. Therefore, we can assume laminar flow condition and use Stokes law to find the magnitude of the drag force $\left(F_{d}\right)$ ,

$$F_{d}=6\pi \eta r_{\text{p}}v$$ (12)

Following the derivation presented in Khalil et al. (2012b), the motion equation of the microparticle can be given as following:

$$F_{m}-F_{d}=ma_{p},$$ (13)

where m and $a_{p}$ are the mass and acceleration of the microparticle, respectively. Substitute with Equation 10 and Equation 12 in Equation 13, one obtains Equation 14:

$$\frac{4}{3}\pi r_{p}^{3}\frac{\chi_{m}}{\mu_{0}\left(1+\chi_{m}\right)}f\left(x\right)I^{2}-6\pi \eta r_{\text{p}}v=ma_{p},$$ (14)

### 2.3 Remarks

• The microparticle is assumed to be submerged in the fluid whereas it is actually floating in the water-to-air boundary layer.

• The study is conducted using an open fluidic reservoir augmented with a virtual fluidic-channel with static obstacles inspired from blood vessels with embolus. As such, the dynamics associated with the navigation through real blood vessels such as wall effects and non-Newtonian nonlinearities are not considered in the derived model.

• A digital microscopic camera is employed in our lab experiments to detect the microparticle position. However, in real experiments, when the particle is used inside the human body, specialized imaging systems have to be considered. This may add substantial challenges for real-time localization but that it is not in the scope of this work.

### 2.4 Path planning using artificial potential field

**FIGURE 5:**
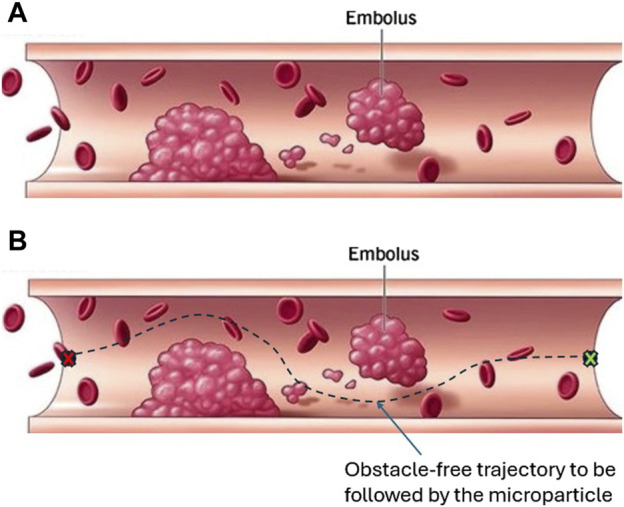
This blood vessel with embolus inspired us to assume a virtual fluidic channel with static obstacles augmented with the open fluidic reservoir as a working environment. **(A)** A vessel with embolus that can block or affect blood circulation. Such an embolus has to be avoided by microparticle when flowing through the blood-vessel. **(B)** For autonomous control, a path planning algorithm is used to generate an obstacle-free trajectory to be tracked by the microparticle Clinic, 2021.

**FIGURE 6:**
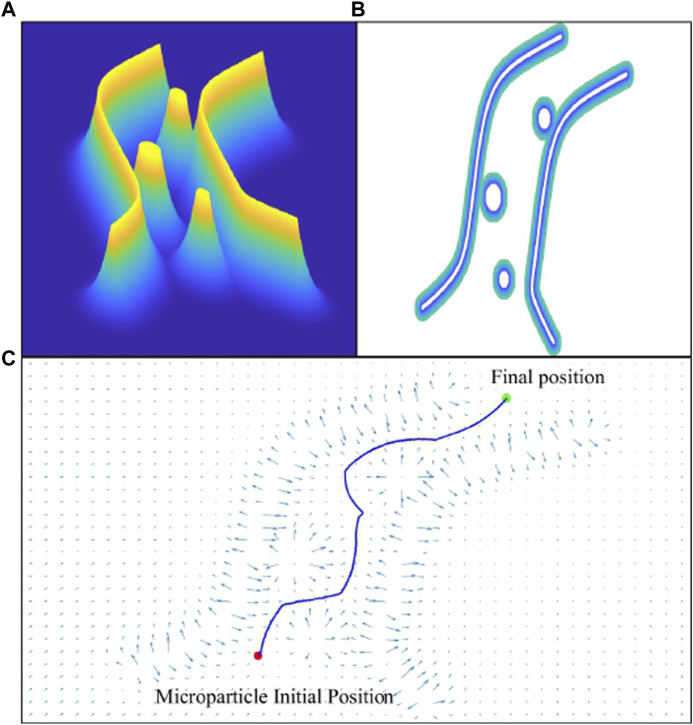
The virtual fluidic channel is represented by a field of attractive and repulsive forces using artificial potential field algorithm. **(A)** Graph of the repulsive potential field. **(B)** 2D view of the fluidic channel and blood clots i.e., obstacles. **(C)** The generated collision-free path from the initial position to final destination of the particle.

This section presents the APF algorithm that generates an obstacle-free path through which the microparticle can reach its destination without collision with the surrounding environment. The microparticle is represented as a point moving under the influence of an attractive potential field generated by the goal and repulsive potential field generated by the obstacles. The direction of the motion of the particle is decided based on the negative gradient of the generated global potential field. The resultant force that drives the particle will be the additive sum of all forces existed due to the gradient of the potential fields. In our case, the particle is assumed to be navigating inside a virtual fluidic channel with a set of obstacles inspired from blood vessels with embolus as shown in Figures 5, 6. To avoid the singularity associated with the canonical form, the attractive potential field of the goal is represented by the quadratic form in Equation 15:

$$U_{att}\left(p\right)=\frac{1}{2}\xi d{\left(p,p_{\text{goal}}\right)}^{2}$$ (15)

where $U_{att}\left(p\right)$ is the attractive potential field of the goal, ξ is a positive scaling factor that modulates the strength of the attractive field, and $d\left(p,p_{\text{goal }}\right)$ is the euclidean distance between the current position of the particle p and its final destination $p_{\text{goal }}$ . The euclidean distance can be estimated using Equation 16:

$$d\left(p,p_{\text{goal }}\right)=\|p-p_{\text{goal}}\|=\sqrt{{\left(x-x_{\text{goal}}\right)}^{2}+{\left(y-y_{\text{goal}}\right)}^{2}}$$ (16)

where x , y are the coordinates of the current position of the particle p , and $x_{\text{goal}}$ , $y_{\text{goal}}$ are the coordinates of the goal position $p_{\text{goal}}$ . The attractive force acting on the particle can be obtained by finding the gradient of the attractive potential field as following in Equation 17:

$$F_{att}\left(p\right)=\nabla U_{att}\left(p\right)=\xi \left(p-p_{\text{goal }}\right)=\xi \left[\begin{array}{c} x-x_{\text{goal}}\\ y-y_{\text{goal}} \end{array}\right]$$ (17)

On the other side, the repulsive potential field that represents the obstacles can be estimated using the following formula of Equation 18:

$$U_{rep}\left(p\right)=\left\{\begin{array}{ccc} & \frac{1}{2}\eta {\left(\frac{1}{D\left(p\right)}-\frac{1}{Q^{*}}\right)}^{2}, & D\left(p\right)\leq Q^{*}\\ & 0, & D\left(p\right)>Q^{*} \end{array}\right.$$ (18)

where $U_{rep}\left(p\right)$ is the repulsive potential field of the obstacles, η is a scaling factor, D(p) the distance between the particle and the closest sensed point on the obstacle, and $Q^{*}$ is the radius of influence of the obstacle i.e., the distance from which the robot begins to feel the presence of the repulsive potential. The repulsive force acting on the particle can be obtained by finding the gradient of the repulsive potential field as following in Equation 19:

$$F_{rep}\left(p\right)=\left\{\begin{array}{ccc} & \eta \left(\frac{1}{Q^{*}}-\frac{1}{D\left(p\right)}\right)\frac{\nabla D\left(p\right)}{D{\left(p\right)}^{2}}, & D\left(p\right)\leq Q^{*}\\ & 0, & D\left(p\right)>Q^{*} \end{array}\right.$$ (19)

The total potential field under which the particle is moving can be obtained by summing the attractive potential of the goal and repulsive potential of the obstacles as following in Equation 20:

$$U_{\mathrm{tot}}\left(p\right)=U_{att}\left(p\right)+U_{rep}\left(p\right)$$ (20)

In our case, the equation of the total potential field has to take into consideration the multiple repulsive potentials due to the three circular obstacles and two edges. The new equation of the total potential can be formulated as following in Equation 21:

$$U_{\mathrm{tot}}\left(p\right)=U_{att}\left(p\right)+\overset{n}{\underset{i=1}{\sum }}U_{rep_{i}}\left(p\right)+\overset{h}{\underset{j=1}{\sum }}U_{edges_{j}}\left(p\right)$$ (21)

where n is the number of obstacles i.e., n=3 , and h is the number of edges i.e., h=2 . The motion of the particle should be in the direction of the negative gradient of the total potential field towards the lower energy configuration. A simple way to generate the intended path of the particle in the global potential field is the gradient descent method as described by the following equation:

$$\begin{array}{c} x_{\mathrm{new}}=x_{\mathrm{old}}-\alpha \cdot \frac{\partial U_{\mathrm{tot}}\left(p\right)}{\partial x}{|}_{x_{\mathrm{old}}},\\ y_{\mathrm{new}}=y_{\mathrm{old}}-\alpha \cdot \frac{\partial U_{\mathrm{tot}}\left(p\right)}{\partial y}{|}_{y_{\mathrm{old}}} \end{array}$$ (22)

where $x_{\mathrm{new}}$ and $y_{\mathrm{new}}$ are the path coordinates in the new iteration, while $x_{\mathrm{old}}$ and $y_{\mathrm{old}}$ are the path coordinates of the previous iteration, and α is the step size between the iterations. The algorithm of Equation 22 will run inside a while-loop until the difference between the new coordinates of the path and the coordinates of the goal is less than or equal a certain threshold $\|x_{\mathrm{new}}-x_{\text{goal}}\|\leq threshold$ and $\|y_{\mathrm{new}}-y_{\text{goal}}\|\leq threshold$ . Figure 6C shows the generated path from the initial position of the particle to final destination while avoiding the collision with the virtual obstacles or edges of the augmented fluidic channel.

### 2.5 Motion control

**FIGURE 7:**
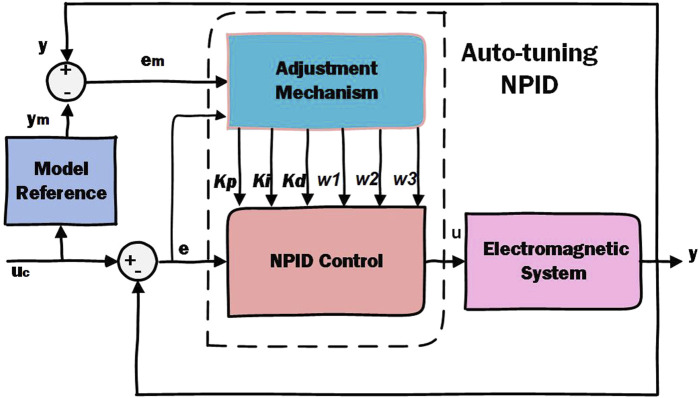
Block diagram of the proposed adaptive nonlinear PID control system. The gains of the A-NPID controller are continuously adjusted by the adaptation mechanism. The equations and parameters of the adaptation mechanism are estimated based on the performance requirements represented by the reference model.

Model Reference Adaptive Controller (MRAC) acts as a servo system with desired performance expressed in form of a reference model. In this work, the NPID control parameters will be adjusted online using the model reference adaptive technique Valluru et al. (2018); Gambier and Yunazwin Nazaruddin (2018); Sirsode et al. (2019). Figure 7 shows the block diagram of the whole system in presence of the proposed control scheme. The reference model is designed such that its output satisfies the performance requirements of our system. In real time, the difference between the ideal output of the reference model and the actual output of the system is sent to an adjustment mechanism that calculates the new gains of the NPID controller as described hereafter, Shamseldin et al. (2022); Shamseldin (2023a); Shamseldin (2023b).The proposed form of the NPID control scheme is given by Equation 23 as following:

$$u\left(t\right)=\left(k_{p}+\tau_{n1}\,\left(e\right)\right)\,\left[e\left(t\right)\right]+\left(k_{i}+\tau_{n2}\,\left(e\right)\right)\cdot \int_{0}^{t}\left[e\left(t\right)\right]\,dt+\left(k_{d}+\tau_{n3}\,\left(e\right)\right)\cdot \left[\frac{de\left(t\right)}{dt}\right]$$ (23)

where $k_{p}$ , $k_{i}$ and $k_{d}$ are the proportional, integral and derivative gains of the linear terms of the control law Equation 23 respectively. As depicted in Figure 7, the linear gains are estimated directly by the adjustment mechanism and sent to the NPID controller. $\tau_{n1}\left(e\right)$ , $\tau_{n2}\left(e\right)$ and $\tau_{n3}\left(e\right)$ are the nonlinear parts of the control law. Each of these non-linear terms is a nonlinear function of two variables; the first variable is the error e between the reference and actual signal, and the second variable is a weight $w_{i}$ estimated by the adjustment mechanism (see Figure 7). Among several functions in the literature, the following form was selected for its simplicity to estimate the non-linear terms of the control law:

$$\tau_{ni}=\frac{exp\left(w_{i}\cdot e\right)+exp\left(-w_{i}\cdot e\right)}{2}$$ (24)

Therefore, the adaptive control law Equation 23 has six adaptation gains that have to be continuously estimated using the adjustment mechanism namely $k_{p}$ , $k_{i}$ , $k_{d}$ , $\tau_{n1}\left(e\right)$ , $\tau_{n2}\left(e\right)$ and $\tau_{n3}\left(e\right)$ . One possible approach to adjust the gains of the model-reference adaptive control law is to follow the MIT rule Astrom and Wittenmark (1994). In this approach, the parameters are adjusted in such a way that the loss function Equation 25 is minimized.

$$J\left(\theta \right)=\frac{1}{2}{e_{m}}^{2}$$ (25)

where J is the loss function that we try to minimize, θ is the adaptation parameter, and $e_{m}$ is the error between the output y of the closed-loop system and the output $y_{m}$ of the reference model that represents the desired closed-loop response. To make J small, it is reasonable to change the parameter in the direction of the negative gradient of J , that is,

$$\frac{d\theta }{dt}=-\gamma \frac{\partial J}{\partial \theta }=-\gamma e_{m}\frac{\partial e_{m}}{\partial \theta }$$ (26)

where γ is a constant assumed by the designer or estimated using an optimization algorithm. When there are many parameters to adjust as in our case i.e. six adaptation parameters, Equation 26 has to be repetitively applied after replacing θ with the parameter of our interest. In this study, the constant γ of the derived adaptation formulas is found using a new effective optimization technique namely COVID-19 Shamseldin (2021). To continue with the derivation of Equation 26, the system model and reference model will be formulated in form of a first order transfer function. For the system model, Equation 14 can be represented as following:

$$a\cdot bF\left(s\right)-cV\left(s\right)=msV\left(s\right),$$ (27)

where $a=\frac{4}{3}\pi r_{p}^{3}\frac{\chi_{m}}{\mu_{0}\left(1+\chi_{m}\right)}$ , $b=0.28\times 10^{5}mT^{2}/m$ is the average value of f(x) at the middle of the reservoir when x=15mm ; note that this approximation is made because the workspace of the particle is assumed to be within a few millimetres from the centre where there is no much change in the value of f(x) . F(s) is the input function and approximated as F(s)=I , $c=6\pi \eta r_{\text{p}}$ , and V(s) is the velocity of the particle. Here is the transfer function representation of Equation 27 shown in Equation 28:

$$TF_{s}\left(s\right)=\frac{V\left(s\right)}{F\left(s\right)}=\frac{ab}{c+ms}=\frac{\frac{ab}{c}}{\frac{m}{c}s+1}=\frac{K}{Ts+1},$$ (28)

where $\frac{ab}{c}$ is the dc gain of the transfer function, while $\frac{m}{c}$ is the time constant of the system. On the other side, the model reference is represented by the following first order transfer where the dc gain $K_{m}$ and time constant $T_{m}$ are assumed such that the response of the model reference is satisfying the requirements of the particle tracking, Equation 29.

$$TF_{m}\left(s\right)=\frac{K_{m}}{T_{m}s+1},$$ (29)

The error $e_{m}$ between the output y of the closed-loop system and the output $y_{m}$ of the reference model can be estimated as following using Equation 30:

$$e_{m}=y-y_{m},$$ (30)

To find the derivative of the adaptation gain $k_{p}$ based on Equation 26, the partial derivative of the error $e_{m}$ with respect to $k_{p}$ is firstly found, Equation 31

$$\frac{\partial e_{m}}{\partial k_{p}}=\frac{K^{2}e}{T_{m}s+1}$$ (31)

Following Equation 26, the derivative of the adaptation gain $k_{p}$ is given by,

$$\frac{dk_{p}}{dt}=-\gamma_{1}e_{m}\frac{K^{2}e}{T_{m}s+1},$$ (32)

Similarly, we can find the adaptation gains $k_{i}$ and $k_{d}$ ,

$$\frac{dk_{i}}{dt}=-\gamma_{2}e_{m}\frac{1}{s}\frac{K^{2}e}{T_{m}s+1},$$ (33)

$$\frac{dk_{d}}{dt}=-\gamma_{3}e_{m}s\frac{K^{2}e}{T_{m}s+1},$$ (34)

The adaptation weights $w_{i}$ of the non-linear terms Equation 24 can be also found in the same way like the adaptation gains Equations 32–34. However, the results showed that even with fixed values for the weights, a satisfactory performance can be achieved. As such, the values of $w_{1}$ , $w_{2}$ , and $w_{3}$ will be estimated once along with the three constants $\gamma_{1}$ , $\gamma_{2}$ , and $\gamma_{3}$ using the optimization algorithm.

## 3 Results

### 3.1 Simulation results

**FIGURE 8:**
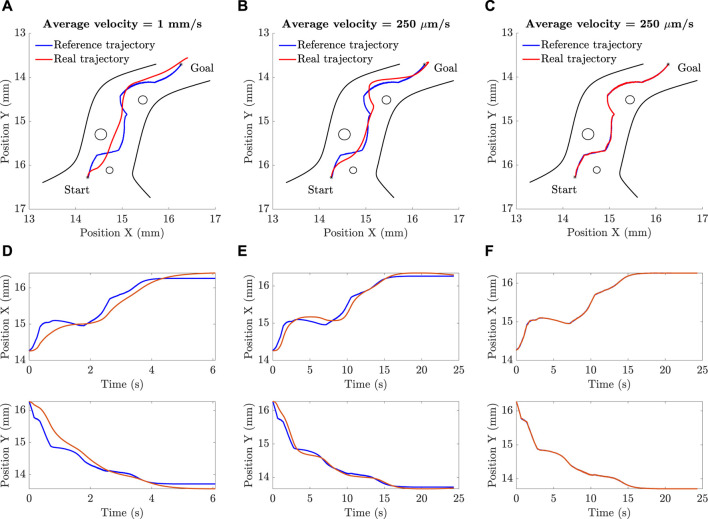
The simulation results show successful navigation of microparticle at different sets of controller parameters. The performance is highly improved when the parameters are estimated using an optimization technique: **(A)**, **(D)** The parameters in this case are selected by trial and error. The time graphs of the x and y positions are created such that the average velocity of the microparticle is 1 mm/s. **(B)**, **(E)** The parameters in this case are selected by trial and error but the average velocity was reduced to 250 μ m/s which considerably enhanced the performance. **(C)**, **(F)** The parameters in this case are selected by an optimization technique which allowed the particle to perfectly track the reference trajectory while moving with an average velocity of 250 μ m/s.

Figure 8 shows the simulation results in which the tracking performance of the microparticle is evaluated. Three tests are conducted at different sets of gains and different sampling rates of the reference data which in turn affects the average velocity of the particle. The collision-free path generated by the artificial potential field planning algorithm in Section 2.3 is used as a reference trajectory. The gains of two tests were selected randomly and then tuned manually. While the gains of the third test were obtained using an optimization algorithm. Figure 8A shows that when the particle is moving fast with an average velocity of 1 mm/s neither the position tracking nor the steady-state error are satisfying. Figure 8D shows the particle tracking in the first test in *x*-axis and *y*-axis where the integral of squared error (ISE) between the reference and actual trajectory in *x*-axis is 0.21 $mm^{2}\cdot s$ while the ISE between the reference and actual trajectory in *y*-axis is 0.31 $mm^{2}\cdot s$ . However, at the same gains of the first test (see Figure 8A) if the sampling rate is reduced causing the average velocity of the particle to be lesss, the performance can be significantly enhanced. Figure 8B shows the position tracking when the average velocity is 250 μ m/s. It shows that due to the reduced average velocity, tracking was improved and the steady state error was less as well. Figure 8E shows the particle tracking in the second test in *x*-axis and *y*-axis where the integral of squared error (ISE) between the reference and actual trajectory in *x*-axis is 0.28 $mm^{2}\cdot s$ while the ISE between the reference and actual trajectory in *y*-axis is 0.21 $mm^{2}\cdot s$ . As expected the ISE in the second test is less than the ISE in the first test taking into consideration that the period of the second test is four times longer than the period of the first test. Figure 8C shows the result of the third test where the gains were obtained using the COVID-19 optimization algorithm. The microparticle could perfectly track the reference trajectory without significant deviation. In addition, the particle could reach its final position with minimal steady-state error. Figure 8F shows the particle tracking in the third test in *x*-axis and *y*-axis where the integral of squared error (ISE) between the reference and actual trajectory in *x*-axis and *y*-axis is nearly zero (about $4e^{-04}\;mm^{2}\cdot s$ ).

### 3.2 Experimental Validation

**FIGURE 9:**
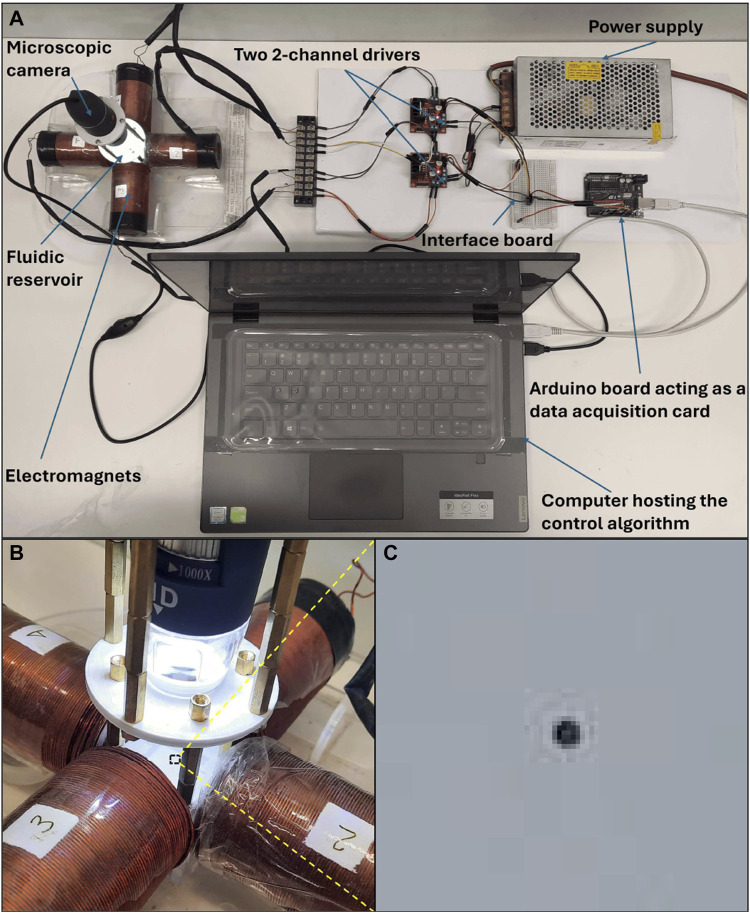
The experimental setup shown in **(A)** has been used to test the proposed A-NPID controller practically. The setup consists of a set of electromagnets, a microscopic camera, two 2-channels drivers,a power supply, an arduino board and PC running a labview VI representing the proposed controller. The arduino board is used as a data acquisition card to send the control signals to the motor drivers. **(B)** The microscopic camera is positioned properly over the target. **(C)** The microparticle is magnified while flowing on the surface of the water in the reservoir.

**FIGURE 10:**
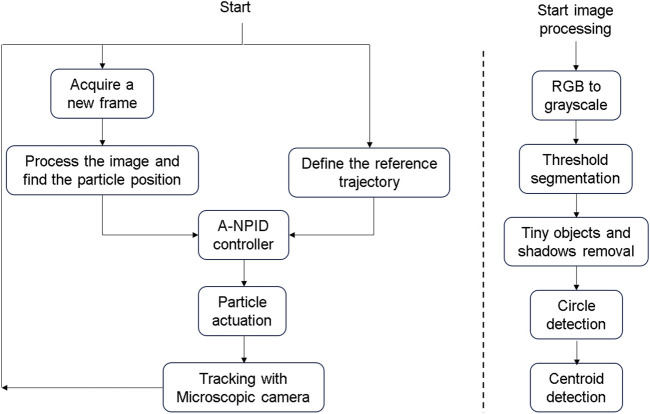
The flowchart on the left-hand side represents the closed loop control system. The controller receives the required and current positions of the particle from the path-planning algorithm and microscopic camera respectively. A control action is taken by the A-NPID and sent to the coils to actuate the particle. The flowchart on the right-hand side represents the operations applied on the image acquired from the microscopic camera to detect the particle position.

The experimental setup consists of four identical electromagnetic coils placed in x-y plane. In order to reduce the coupling effect between the coils, the current direction in all coils should be the same. Having different directions of the current showed that the coupling could significantly reduce the generated magnetic field. Each coil has 1,400 turns of 0.7 mm round copper wire coated with enamel. The inner radius of the coil $\left(r_{i}\right)$ is 10 mm, the outer radius $\left(r_{o}\right)$ is 19.8 mm, the length of the core $\left(l_{c}\right)$ is 80 mm, and the axial length of the windings $\left(l_{w}\right)$ is 70 mm. The coil cores are made of ferromagnetic material i.e., low carbon steel with relative permeability $\mu_{r}$ of around 100. The four coils are driven by two 2-channels drivers of L298N model that allows maximum current of 2A as shown in Figure 9. A PC based control strategy is adopted and the control algorithm is developed as a VI in the LABVIEW environment. The PC used in the experiments has intel processor of Core i7 - 8th generation. The particle position is detected using a microscopic camera with up to 1,000x magnification level. The camera was firstly calibrated to find a relation between the image pixels and real dimensions in mm. The readings of the camera are sent to the PC via USB connection with sampling rate between 25 and 30 frames per second. An arduino board of type UNO is used as an interface between the computer and drivers. For this purpose, an interface library was firstly to the labview to allow communication with arduino toolkit. Figure 10 shows the flowchart of the control algorithm as it starts by acquiring a new frame from the camera and then image processing is applied to detect the position of the microparticle. The A-NPID controller compares the actual position of the particle with the reference trajectory obtained from the path planning algorithm of artificial potential field. The control signal is sent to the drivers through the arduino board to actuate the particle accordingly with the electromagnets. The particle is continuously tracked by the microscopic camera and the loop is repeated until reaching the targeted goal.

**FIGURE 11:**
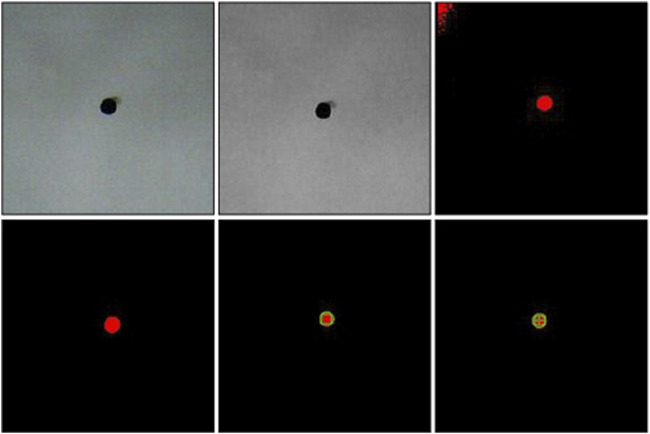
The image acquired from the microscopic camera is processed to detect the particle position. Firstly, the RGB image is converted into a greyscale image. Secondly, a threshold is used to obtain a binary image showing the particle on a black background. Afterwards, the noise is removed and the particle center is detected.

**FIGURE 12:**
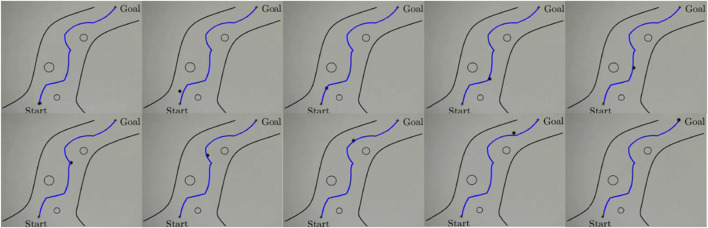
Selected frames from the real experiments augmented with the generated collision-free trajectory along with the virtual fluidic channel and obstacles. The experiment shows successful navigation and arrival at the targeted position. In this example the average velocity of the microparticle is 35 μ m/s.

**FIGURE 13:**
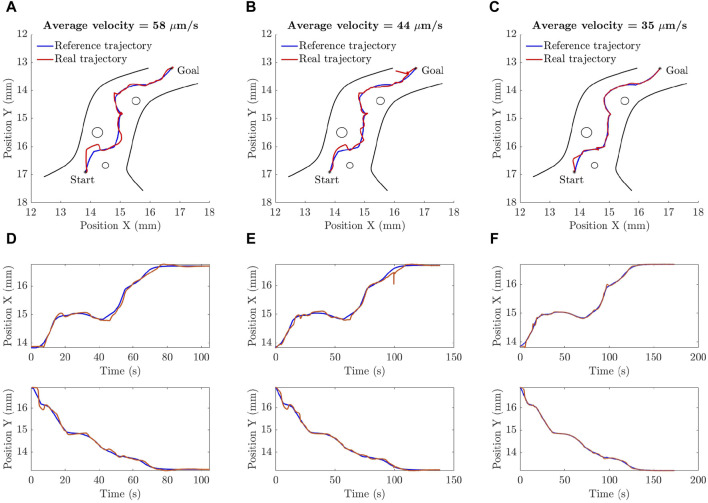
The experimental results verify the ability of the proposed A-NPID controller to drive the microparticle along the required trajectory at different operating conditions. Three tests were conducted at the same set of parameters but with different sampling rate. In all cases, the particle reached its targeted position but deviation that depends on the desired average velocity. **(A)**, **(D)** The particle is moving with an average velocity of 58 μ m/s as several overshoots were observed. **(B)**, **(E)** The particle is moving slightly slower with an average velocity of 44 μ m/s which led to performance enhancement. **(C)**, **(F)** The particle is moving with a reduced average velocity of 35 μ m/s which further improved the tracking.

On the right hand side of Figure 10, there exist another flowchart about the steps of the process of the image processing. After defining the region of interest (ROI), the acquired RGB image is converted into a greyscale image. In the second step, a threshold value is used to get a binary image where the contrast between the particle and background is clear. In the third step the tiny objects that has smaller diameters than the particle are removed along with the shadows near the edges of the image. In the fourth step, the outer circumference of the particle is recognized as a circle. Then, its radius is estimated in pixels and compared with the previously known diameter of 100 μ m. This process is used to calibrate the microscopic camera and find a relation between the pixels of the acquired images and real dimensions in mm. In the final step, the centroid of the particle is determined and sent to the controller as the measured position of the particle. Figure 11 shows the different operations applied on the image of the particle.The proposed ANPID control algorithm was verified experimentally as well. Figure 12 shows some selected frames of the microparticle while following the reference trajectory. Three tests were conducted at three different average velocities. The results showed that the ANPID control algorithm is capable to achieve successful tracking in all cases as the microparticle could navigate without the collision with any of the obstacles till reaching the final destination. However, the results show that the average velocity of the particle has significant effect on the tracking performance. For instance, in Figure 13A the particle could track the reference trajectory and reach its goal but with multiple deviation from the reference due to the relatively high average velocity of about 58 μ m/s Figure 13D shows the particle tracking in the first test in *x*-axis and *y*-axis where the ISE between the reference and actual trajectory in *x*-axis is 0.43 $mm^{2}\cdot s$ while the ISE between the reference and actual trajectory in *y*-axis is 0.56 $mm^{2}\cdot s$ . The test was repeated at lower velocities and the results showed that the lower the velocity the better the tracking when the same set of gains are used. Figure 13B shows the tracking of the microparticle at an average velocity of 44 μ m/s where less deviation is observed compared to the graph of Figure 13A. Figure 13E shows the particle tracking in the second test in *x*-axis and *y*-axis where the ISE between the reference and actual trajectory in *x*-axis is 0.53 $mm^{2}\cdot s$ while the ISE between the reference and actual trajectory in *y*-axis is 0.4 $mm^{2}\cdot s$ . The result of the third test is shown in Figure 13C where the average velocity is about 35 μ m/s. The tracking in this case was highly improved due to the reduced velocity of the particle. Figure 13F shows the particle tracking in the third test in *x*-axis and *y*-axis where the ISE between the reference and actual trajectory in *x*-axis is 0.15 $mm^{2}\cdot s$ while the ISE between the reference and actual trajectory in *y*-axis is 0.19 $mm^{2}\cdot s$ . The slight incease in ISE of the second test over the ISE of the first test is due to conducting the experiment for longer time; four times of the first experiment causing the accumulated squared error to be slightly increased. However, the trend of all ISE values shows the improvement in the performance.

**FIGURE 14:**
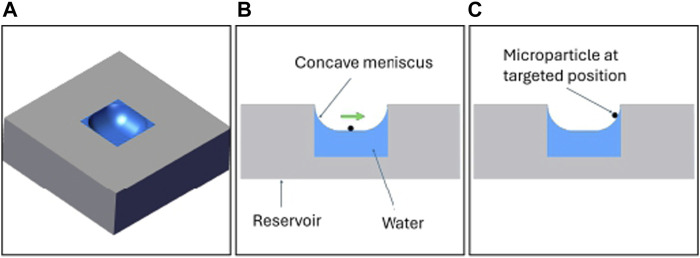
A concave meniscus occurs when the molecules of the water are attracted to those of the container. The operating conditions changes from the middle of the reservoir than the right side of the reservoir due to the inclination of the water surface.

**FIGURE 15:**
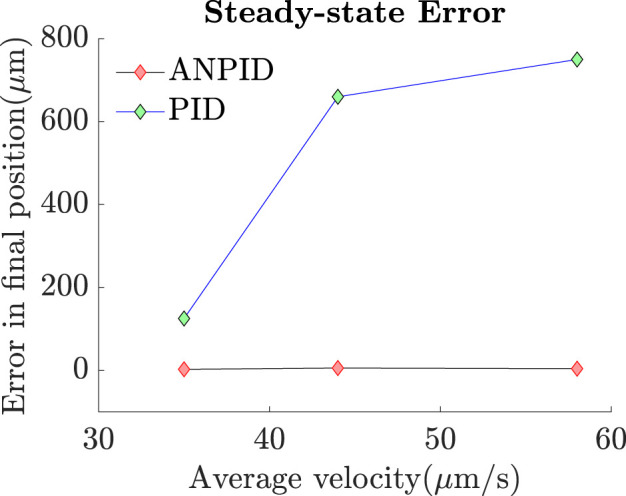
The A-NPID controller allowed the microparticle to reach its targeted position with minimal steady-state error even in presence of concave meniscus. However, in case of using the PID controller in absence of the adaptation terms, the particle could successfully track the trajectory but failed to reach its final destination with minimal steady-state error due to the sudden inclination of water surface.

The same tests were repeated in absence of the adaptive terms of the A-NPID control law as only the PID gains were left. The results showed that the PID controller allowed the particle to track the reference trajectory with obstacles collision avoidance but with significant deviation and steady state error. The reason behind such a degradation in the performance is that the final destination is near the edge of the reservoir where the surface of the water is not as flat as in the middle of the reservoir. Figure 14 depicts this phenomenon of concave meniscus which takes place due to the surface tension and adhesion force between the water and reservoir. Such a parabolic inclination represents different environmental conditions that requires tuning the gains of the controller. Since the linear PID control law has fixed gains, the performance was degraded when the particle approached the inclined surface. Figure 15 compares the performance of both controllers, A-NPID and PID, in terms of steady state error. The graph shows superiority of A-NPID due to its adaptation mechanism that allows the gains to be continuously adjusted in order to satisfy the performance requirements represented by the model reference. It is shown that at all operating conditions, the steady-state error was as small as 4 μ m. This value changes slightly within the range of 2 or 3 μ m when the average velocity increases. On the other side, when the PID is used the steady state error reached in the best case to about 150 μ m due to the nonlinear parabolic inclination near the edge of the reservoir.

## 4 Discussion and conclusions

In this work, an adaptive nonlinear PID control scheme was proposed for autonomous navigation and control of microparticles. The proposed controller allowed the gains to be continuously adjusted to cope with the varying operating conditions and to satisfy the performance requirements. The results showed that the A-NPID was able to drive the microparticle successfully to follow a collision-free trajectory and reach its destination with minimal steady-state error of about 4 μ m. A slight increase in the steady-state error of about 2 to 3 μ m and a degradation in the tracking performancewas were observed when the required average velocity of the microparticle increased. The reference trajectory was generated using the artificial potential field algorithm. The proposed A-NPID was put in comparison with the traditional PID controller where a degradation in the performance in terms of steady-state error was observed when the adaptive term was not used. The reason was that the PID gains could not behave efficiently at all operating points specially when the targeted position is placed near a concave meniscus. In this work, a complete study was presented. Firstly, the mathematical model of the electromagnetic system was derived. Then, the artificial potential field path planning algorithm was used to generate a collision-free trajectory through an open fluidic reservoir with virtual static obstacles. The generated trajectory was used as a reference signal to the control system. The derived mathematical model was then used to design the control system and find the corresponding gains. All the results were verified experimentally and by simulation.

## 5 Future work

In future studies, the motion control presented in this paper will be put in comparison with other advanced control algorithms such as Model Predictive Control (MPC), Fuzzy Logic Control, or Reinforcement Learning-based controllers. This would highlight the relative strengths and weaknesses of the proposed approach. In addition, exploring adaptive mechanisms to further tune the controller parameters in real-time based on the changing dynamics of the environment should be considered. It is also necessary to employ a variety of test scenarios for more comprehensive evaluation of the proposed controller’s robustness.

## Data Availability

The original contributions presented in the study are included in the article/supplementary material, further inquiries can be directed to the corresponding author.
